# Conservatively treated Congenital Hyperinsulinism (CHI) due to K-ATP channel gene mutations: reducing severity over time

**DOI:** 10.1186/s13023-016-0547-3

**Published:** 2016-12-01

**Authors:** Maria Salomon-Estebanez, Sarah E. Flanagan, Sian Ellard, Lindsey Rigby, Louise Bowden, Zainab Mohamed, Jacqueline Nicholson, Mars Skae, Caroline Hall, Ross Craigie, Raja Padidela, Nuala Murphy, Tabitha Randell, Karen E. Cosgrove, Mark J. Dunne, Indraneel Banerjee

**Affiliations:** 1 Department of Paediatric Endocrinology, Royal Manchester Children’s Hospital, Central Manchester University Hospitals, Oxford Road, Manchester, M13 9WL UK; 2 Institute of Biomedical and Clinical Science, University of Exeter Medical School, RILD Building, RD&E Hospital Wonford, Barrack Road, Exeter, EX2 5DW UK; 3 Department of Paediatric Endocrinology and Diabetes, Nottingham Children’s Hospital, Nottingham University Hospitals, Derby Road, Nottingham, NG7 2UH UK; 4 Paediatric Psychosocial Department, Royal Manchester Children’s Hospital, Central Manchester University Hospitals, Oxford Road, Manchester, M13 9WL UK; 5 Therapy and Dietetic Department, Royal Manchester Children’s Hospital, Central Manchester University Hospitals, Oxford Road, Manchester, M13 9WL UK; 6 Department of Paediatric Surgery, Royal Manchester Children’s Hospital, Central Manchester University Hospitals, Oxford Road, Manchester, M13 9WL UK; 7 Department of Diabetes and Endocrinology, Children’s University Hospital, Temple Street, Dublin, Ireland; 8 Faculty of Biology, Medicine and Health, University of Manchester, Oxford Rd, Manchester, M13 9PL UK

**Keywords:** Hypoglycaemia, Congenital hyperinsulinism, Insulin, Genetics, Mutations, Diazoxide, Octreotide, Neurodevelopment

## Abstract

**Background:**

Patients with Congenital Hyperinsulinism (CHI) due to mutations in K-ATP channel genes (K-ATP CHI) are increasingly treated by conservative medical therapy without pancreatic surgery. However, the natural history of medically treated K-ATP CHI has not been described; it is unclear if the severity of recessively and dominantly inherited K-ATP CHI reduces over time. We aimed to review variation in severity and outcomes in patients with K-ATP CHI treated by medical therapy.

**Methods:**

Twenty-one consecutively presenting patients with K-ATP CHI with dominantly and recessively inherited mutations in *ABCC8/KCNJ11* were selected in a specialised CHI treatment centre to review treatment outcomes. Medical treatment included diazoxide and somatostatin receptor agonists (SSRA), octreotide and somatuline autogel. CHI severity was assessed by glucose infusion rate (GIR), medication dosage and tendency to resolution. CHI outcome was assessed by glycaemic profile, fasting tolerance and neurodevelopment.

**Results:**

CHI presenting at median (range) age 1 (1, 240) days resolved in 15 (71%) patients at age 3.1(0.2, 13.0) years. Resolution was achieved both in patients responsive to diazoxide (*n* = 8, 57%) and patients responsive to SSRA (*n* = 7, 100%) with earlier resolution in the former [1.6 (0.2, 13.0) v 5.9 (1.6, 9.0) years, *p* = 0.08]. In 6 patients remaining on treatment, diazoxide dose was reduced in follow up [10.0 (8.5, 15.0) to 5.4 (0.5, 10.8) mg/kg/day, *p* = 0.003]. GIR at presentation did not correlate with resolved or persistent CHI [14.9 (10.0, 18.5) v 16.5 (13.0, 20.0) mg/kg/min, *p* = 0.6]. The type of gene mutation did not predict persistence; resolution could be achieved in recessively-inherited CHI with homozygous (*n* = 3), compound heterozygous (*n* = 2) and paternal mutations causing focal CHI (*n* = 2). Mild developmental delay was present in 8 (38%) patients; adaptive functioning assessed by Vineland Adaptive Behavior Scales questionnaire showed a trend towards higher standard deviation scores (SDS) in resolved than persistent CHI [−0.1 (−1.2, 1.6) v −1.2 (−1.7, 0.03), *p* = 0.1].

**Conclusions:**

In K-ATP CHI patients managed by medical treatment only, severity is reduced over time in the majority, including those with compound heterozygous and homozygous mutations in *ABCC8/KCNJ11.* Severity and treatment requirement should be assessed periodically in all children with K-ATP CHI on medical therapy.

**Electronic supplementary material:**

The online version of this article (doi:10.1186/s13023-016-0547-3) contains supplementary material, which is available to authorized users.

## Background

Congenital Hyperinsulinism in Infancy (CHI) is a rare disorder causing severe debilitating hypoglycaemia, usually presenting in infancy [1, 2]. Hypoglycaemia due to CHI can have a deleterious impact on early life brain function, with several cohorts reporting adverse neurodevelopmental outcomes in a third to a half of patients [3–6]. The frequency of hypoglycaemia-related brain injury in the CHI population as a whole has not reduced despite optimisation of diagnosis and treatment over the last decade. The burden of morbidity in CHI continues to be a major problem for individuals and health care professionals; therefore, a greater focus is required on understanding variations in disease severity.

Genetic understanding of CHI has progressed rapidly with a significant proportion of CHI found to have underlying genetic causes, most frequently mutations in the K-ATP channel genes, *ABCC8* and *KCNJ11* [7, 8]. K-ATP channel genotyping has stratified treatment protocols of focal and diffuse CHI with paternal heterozygosity most commonly associating with focal CHI and maternal heterozygous, homozygous or compound heterozygous mutations in *ABCC8*/*KCNJ11* associating with diffuse disease [2]. Although paternal heterozygosity has a higher predilection for focal CHI, additional investigation such as 18-fluoro-dopa PET-CT scanning is necessary to localise the lesion in focal CHI; a significant proportion, as many as half with paternal heterozygous mutations in some reports may have diffuse CHI [9] which could be explained by dominant inheritance or inability to identify a maternal mutation in recessively-inherited disease.

It is recognised that pancreatectomy, either lesionectomy for focal lesions or subtotal pancreatectomy for severe diffuse CHI is a well-established treatment choice for CHI. However, increasingly there is a shift to conservative medical management particularly in the case of diffuse CHI which is traditionally treated by near total pancreatectomy. Indeed, some children with focal CHI in the head of the pancreas proximal or abutting the bile duct may benefit from conservative treatment due to the nature of the surgical complexity involved. In our centre, the frequency of patients (with K-ATP and non-K-ATP channel gene mutations) undergoing pancreatic surgery as a proportion of new patients referred to the service has reduced from 18% in 2007–2008 to 6–7% in 2014–2015.

A number of case reports of spontaneous resolution of disease have been reported [10–12], mostly in those without known genetic mutations, while cohort studies in different countries have characterised surgical outcomes only [4, 7, 8, 13]. Long-term conservative treatment with diazoxide and octreotide without requirement for pancreatic surgery has also been reported in patients with and without K-ATP channel gene mutations [12, 14, 15]; however these observations do not offer insight into the evolution of disease severity and if treatment response improves or worsens over time. Therefore, disease trajectories of medically treated K-ATP CHI remain poorly understood. It is important to understand the trends in severity of CHI to modify and individualise the intensity of medical therapy. Here we have studied a cohort of patients with K-ATP CHI treated by medical therapy to examine outcomes of disease in follow up assessments.

## Methods

The aims of our study were to assess variation in intensity of treatment in children with K-ATP CHI over time, and to review outcomes of medically treated K-ATP CHI patients in follow up assessments.

A cohort of patients with K-ATP CHI (mutations in *ABCC8/KCNJ11*) treated by medical therapy (*n* = 21) was identified from a group of patients (*n* = 404) in a specialist centre for CHI between April 2006 and July 2016, with local Research Ethics approval. Genetic investigations were performed in 269 patients only within the cohort. In the remainder, genetic investigations were not performed because CHI resolved in early infancy or patients remained on low dose diazoxide. In those undergoing genetic testing, 71 patients had mutations in *ABCC8/KCNJ11*, 10 patients had mutations in other genes related to CHI (*HNF4A, GCK, HADH, GLUD1*) and 10 patients had variants of uncertain clinical significance. Within the group of 71 patients with *ABCC8/KCNJ11* mutations, 39 patients underwent pancreatic surgical treatment (subtotal pancreatectomy or focal lesionectomy); patients who were not surgically treated, i.e. medically treated (*n* = 21) were recruited to the study. Eleven patients who were also medically treated were not recruited because they either presented between January 2016 and July 2016, or insufficient clinical information was available in follow up.

The diagnosis of CHI was made in patients presenting to this centre using well-established criteria [1, 2]. Patients underwent rapid K-ATP channel gene mutation analysis as per protocol, as previously reported [10]. Variants either previously reported or considered likely to be pathogenic were included in the cohort. One variant reported as pathogenic in our patient but classified elsewhere as being a variant of uncertain significance was also included.

The diagnosis of focal CHI was made on the basis of a paternal heterozygous mutation in *ABCC8/KCNJ11* and confirmed by identification of a solitary lesion in the pancreas during 18-fluoro-dopa PET-CT scanning [2]. Those with no clear foci were diagnosed as diffuse CHI. Diffuse CHI was also presumed if the patient had maternal heterozygous, homozygous, or compound heterozygous mutations in *ABCC8/KCNJ11*, for which 18-fluoro-dopa PET-CT scans were not performed. Patients with *ABCC8/KCNJ11* mutations who required either lesionectomy for focal CHI or subtotal pancreatectomy for diffuse CHI were excluded from the cohort. Patients who underwent pancreatic biopsy or minimal resection while continuing medical therapy were included in the cohort.

Treatment variations were made on clinical grounds and individualised to patient need. Oral diazoxide was used as first line treatment, while somatostatin agonists (SSRA, octreotide, somatuline) were used as second line treatment. Carbohydrate supplements to increase energy content of milk and polyunsaturated fatty acids (PUFA) used in the management of diazoxide responsive CHI were considered as food supplements and did not preclude inclusion to the cohort [16]. The dose of Eicosapentaenoic acid (EPA) component of omega-3 fatty acid was allowed in a range of 240–480 mg per day. Responsiveness to diazoxide as treatment for CHI was determined by noting satisfactory glucose profiling and fasting tolerance as described previously [16]. Responsiveness to SSRA was also determined in a similar manner.

Children had resolution of CHI if treatment was minimised and withdrawn completely with maintenance of satisfactory glucose profiles (95% values >3.5 mmol/L) on home glucose monitoring or subcutaneous continuous glucose monitoring (CGM) [10, 16]. To achieve resolution of CHI, satisfactory fasting tolerance was mandatory with end of fast blood glucose >3.0 mmol/L, suppressed insulin concentrations and blood ketones >1.0 mmol/L measured by point of care testing and/or laboratory analysis of 3 hydroxybutyrate. Follow-up consisted of telephone reviews every 2 weeks for the first 4 months, followed by clinic reviews at 4 monthly intervals by a multi-disciplinary team including a clinician, two specialist nurse practitioners, two dietitians, a speech and language therapist and a clinical psychologist. At each review, glucose profile was assessed and medication adjusted accordingly. Children who demonstrated resolution of CHI were reviewed in clinic appointments every 6 months by a clinician and specialist nurse practitioner without wider multi-disciplinary team input. Annual home blood glucose profiles were assessed to determine glycaemic status and to ensure continuing euglycaemia. Oral glucose tolerance testing was not performed routinely in all children undergoing spontaneous resolution, in the absence of information regarding long-term utility and difficulty in administering the test in young children. Instead, home blood glucose profiling was assessed and correlated with symptoms of hypoglycaemia and hyperglycaemia. Pancreatic biopsy was not routinely undertaken in patients enrolled in the cohort. However, for patients in whom a pancreatic biopsy was undertaken as a partial resection, the tissue was analysed for characteristics of focal and diffuse CHI [17].

In addition to glycaemic outcomes in follow up assessment, the Vineland Adaptive Behavior Scales, version II (VABS-II), a questionnaire completed by parents was used to assess adaptive functioning in the domains of communication, daily living skills, social skills and motor skills after age 1.5 years (http://www.pearsonclinical.com/). Information was also obtained on the prevalence of seizures and delayed development in clinical assessment [3]. Auxology parameters were reviewed at the 2 year follow up assessment and measurements were converted to Standard Deviation Scores (SDS) [18]. Statistical analysis was performed by IBM-SPSS version 23.0 (IBM incorporated, New York, USA); Mann–Whitney test was performed to test differences between non-parametric independent variables while paired t-tests were used to test difference between paired samples.

## Results

### Patient characteristics

Twenty-one patients presented with hypoglycaemia at a median age (range) 1 day(1 day, 8 months) with glucose 1.7(0.1, 2.6) mmol/L, insulin 97.2(16.8, 234.0) pmol/L and glucose infusion rate 14.9(10.0, 20.0) mg/kg/min. Birth weight SDS was 2.0(−0.5, +3.8), with weight SDS and height SDS at age 2 years being +1.7(−1.4, +3.8) and +1.0(−2.0, +2.2) respectively. Information on age at presentation, focal and diffuse CHI, medication, feeding and neurodevelopment has been provided in Table 1 with gene mutation status provided in Table 2.

**Table 1:** Patient characteristics

Patient	Current Age (years)	Age at presentation	Resolved at (years)	Focal/Diffuse	Mutation	Maximum Medication dose	Current Medication dose	Feeding method	Neurodevelopment
#1	5.3	Neonate	2.6	Diffuse	Compound heterozygous *ABCC8*	DZX 5 mg/kg/d	0	Orally	Speech delay
#2	9.3	Neonate	3.5	Diffuse	Maternal *KCNJ11*	DZX 10 mg/kg/d	0	Gastrostomy (4 years)	Speech delay
#3	16.6	Neonate	13	Diffuse	*de novo ABCC8*	DZX 7 mg/kg/d	0	Orally	Normal
#4	9.4	Neonate	0.7	Diffuse	Maternal *ABCC8*	DZX 9.2 mg/kg/d	0	Orally	Seizures at presentation, behavioural problems
#5	4.3	Neonate	3.1	Diffuse	Maternal *ABCC8*	DZX 7.1 mg/kg/d	0	Orally	Normal
#6	0.7	Neonate	0.4	Diffuse	Paternal *ABCC8*	DZX 5 mg/kg/d	0	Orally	Normal
#7	6.7	Day 2	0.5	Diffuse	Maternal *ABCC8*	DZX 5 mg/kg/d	0	Orally	Epilepsy, motor delay, coordination problems
#8	2.7	Day 2	0.2	Diffuse	Maternal *ABCC8*	DZX 5 mg/kg/d	0	Orally	Normal
#9	7.2	Day 1	6	Diffuse	Homozygous *ABCC8*	OCT 18.5 mcg/kg/d; Somatuline 60 mg 4-7 weekly	0	Gastrostomy (2.5 years)	Normal
#10	2.7	Day 1	1.6	Focal	Paternal *KCNJ11*	OCT 15 mcg/kg/d	0	Gastrostomy (1.3 years)	Normal
#11	10.9	8 months	6.6	Focal	Paternal *ABCC8*	OCT 19 mcg/kg/d	0	Orally	Normal
#12	7.1	Day 1	7	Diffuse	Compound heterozygous *ABCC8*	OCT 14.5 mcg/kg/d	0	Gastrostomy (3.6 years)	Normal
#13	5.7	Day 1	1.9	Diffuse	Paternal *KCNJ11*	OCT 3.8 mcg/kg/d	0	Gastrostomy (1.7 years)	Normal
#14	12.3	Day 1	9	Diffuse	Homozygous *ABCC8*	OCT 19.2 mcg/kg/d	0	Gastrostomy (1.2 years)	Mild gross motor, speech delay
#15	7.6	Day 70	5.5	Diffuse	Presumed paternal *KCNJ11*	OCT 17 mcg/kg/d	0	Gastrostomy (2.0 years)	Normal
#16	8.9	Day 5	Not resolved	Diffuse	Paternal *KCNJ11*	DZX 10 mg/kg/d	DZX 6 mg/kg/d	Orally	Epilepsy, speech, motor, learning difficulties
#17	1.1	Day1	Not resolved	Diffuse	Homozygous *ABCC8*	DZX 10 mg/kg/d	DZX 0.5 mg/kg/d	Orally	Normal
#18	1.3	Day 2	Not resolved	Diffuse	Paternal *KCNJ11*	DZX 8.5 mg/kg/d	DZX 5.8 mg/kg/d	Orally	Normal
#19	5.1	Neonate	Not resolved	Diffuse	*de novo ABCC8*	DZX 15 mg/kg/d	DZX 10.8 mg/kg/d	Gastrostomy (continuing at present)	Normal
#20	6.8	Day 2	Not resolved	Diffuse	Maternal *ABCC8*	DZX 9.6 mg/kg/d	DZX 3.1 mg/kg/d	Orally	Epilepsy, motor delay, behavioural problems
#21	3.2	Day 1	Not resolved	Diffuse	Paternal *KCNJ11*	DZX 12.5 mg/kg/d	DZX 5 mg/kg/d	Gastrostomy (overnight only, continuing)	Speech delay

Patient characteristics in this cohort of patients with medically treated K-ATP CHI (*n* = 21), showing age at presentation, resolution status, diffuse/focal, medication dosage (DZX - diazoxide, OCT - octreotide), feeding practices and neurodevelopmental status. The type of genetic mutation (see also Table 2) has no correlation with resolution status of CHI. The mutation in patient #16 has also been classified as a variant [8]

**Table 2:** Genetic characterisation of patients with medically treated K-ATP CHI

Patient	Mutation	Paternal	Maternal	*de novo*	Reference
#1	Large deletion/Missense	*ABCC8* p.? (c.(2258+1_2259-1)_(2294+1_2295-1)del)	*ABCC8* p.A355T (c.1063G>A)		Deletion is novel, A355T reported in Ismail (2010) [24], Russo (2011) [25], Snider (2013) [8], Mohnike (2014) [26]
#2	Missense		*KCNJ11* p.T294M (c.881C>T)		Arya (2014) [9], Shimomura (2009) [27], Bellanne-Chantelot (2010) [28], Ilmaran (2010) [29], Gong (2015) [13]
#3	Missense			*ABCC8* p.L508P (c.1523T>C)	Aguilar-Bryan (1999) [30]
#4	Missense		*ABCC8* p.T1516M (c.4547C>T)		Banerjee (2011) [10]
#5	Missense		*ABCC8* p.R1539Q (c.4616G>A)		Pinney (2008) [31], Park (2011) [32], Kapoor (2011) [33]
#6	Missense	*ABCC8* p.A1263T (c.3787G>A)			Ayra (2014) [9], Christesen (2012) [34]
#7	Missense		*ABCC8* p.G1382S (c.4144G>A)		Nestorowicz (1998) [35], Shyng (1998) [36]
#8	Missense		*ABCC8* p.T1516M (c.4547C>T)		Banerjee (2011) [10]
#9	Splicing/Splicing	*ABCC8* p.? (c.1467+5G>A)	*ABCC8* p.? (c.1467+5G>A)		Powell (2011) [37]
#10	Missense	*KCNJ11* p.R34C (c.100C>T)			Snider (2013) [8]
#11	Splicing	*ABCC8* p.? (c.2041-21G>A)			Ohkubo (2005) [38], Suchi (2006) [39], Hardy (2007) [40], Mohnike (2014) [26], Lee (2015) [41]
#12	Missense/Nonsense	*ABCC8* p.G70R (c.208G>A)	*ABCC8* p.R842* (c.2524G>T)		G70R: Banerjee (2011) [10]; R842* : Brunetti-Pierri (2008) [42], Mohnike (2014) [26]
#13	Missense	*KCNJ11* p.G40D (c.119G>A)			Suchi (2006) [39]
#14	Splicing/Splicing	*ABCC8* p.? (c.1467+5G>A)	*ABCC8* p.? (c.1467+5G>A)		Powell (2011) [37]
#15	Missense	*KCNJ11* p.G40D (c.119G>A)*			Suchi (2006) [39]
#16	Missense	*KCNJ11* p.R195H (c.584G>A)			Coventry (2010) [43], Russo (2011) [25], Snider (2013) [8]
#17	Missense/Missense	*ABCC8* p.R526C (c.1576C>T)	*ABCC8* p.R526C (c.1576C>T)		Sogno Valin (2013) [21], Snider (2013) [8], Arya (2014) [44]
#18	Missense	*KCNJ11* p.R206L (c.617G>T)			Novel
#19	Missense			*ABCC8* p.I1512T (c.4535T>C)	Pinney (2008) [31]
#20	Missense		*ABCC8* p.D310N (c.928G>A)		Fernandez-Marmiesse (2006) [45], Pinney (2008) [31]
#21	Missense	*KCNJ11* p.R206C (c.616C>T)			Bennett (2015) [46]

Genetic characterisation of patients with medically treated K-ATP CHI, showing gene defect, protein changes, type of mutation, mode of inheritance and citations (see references). *The p.G40D mutation is presumed to be of paternal origin. The mutation was not present in the sample from the mother and the father was unavailable for testing. The mutation in patient #16 has been classified in other publications as a variant of uncertain significance

Recessively acting mutations were identified in 7 (33%) patients; 3 patients had homozygous mutations, 2 patients had compound heterozygous mutations in *ABCC8* and 2 patients had focal CHI (one paternally inherited mutation in *ABCC8* and one paternally inherited mutation in *KCNJ11*). A single heterozygous mutation was identified in 14 (67%) patients; 5 patients had maternally inherited *ABCC8* mutations, 2 patients had *de novo ABCC8* mutations (no mutations identified in parents), 1 patient had a paternally inherited *ABCC8* mutation without focal CHI, 5 patients had paternally inherited *KCNJ11* mutations without focal CHI and 1 patient had a maternally inherited *KCNJ11* mutation.

### Case illustrations

1. Patient #9 with a homozygous *ABCC8* mutation and severe CHI at presentation was unresponsive to diazoxide. He was treated with octreotide via subcutaneous pump to a maximum dose of 18.5 mcg/kg/day and then switched to somatuline autogel 60 mg every 4 weeks subcutaneously. Monitoring at home showed normal glucose profiles, prompting somatuline injection intervals to be gradually increased from 4 to 7 weeks without recurrence of hypoglycaemia. However, the patient became increasingly intolerant of needles and injections, at which point his parents requested a trial period without medical therapy, adding PUFA as a food supplement and monitoring carefully for relapse into hypoglycaemia. One year after stopping somatuline, this patient remains on PUFA as a food supplement in a dose 260 mg twice a day with satisfactory fasting tolerance, normal food frequency and regular daily activity including school.
2. Patient #10 with a previously reported paternal *KCNJ11* missense mutation and 18- fluoro-dopa PET-CT scanning suggesting a lesion in the tail also had severe CHI at presentation. In the pre-operative period, euglycaemia was achieved with a combination of octreotide in a dose of 15 mcg/kg/day and gastrostomy feeding. At laparoscopic surgery, the lesion was not identified at the anatomical location suggested by imaging investigations. Her pancreatic tail biopsy showed normal histology, implying the presence of focal CHI elsewhere in the pancreas. Following discussion with parents, she was medically-treated with octreotide. In follow up, octreotide was gradually decreased and then stopped at age 1.6 years with satisfactory fast tolerance and normal glucose profiles, which persists after 1.1 years of treatment withdrawal.

### Variation in natural history: tendency to resolution

**Fig. 1:**
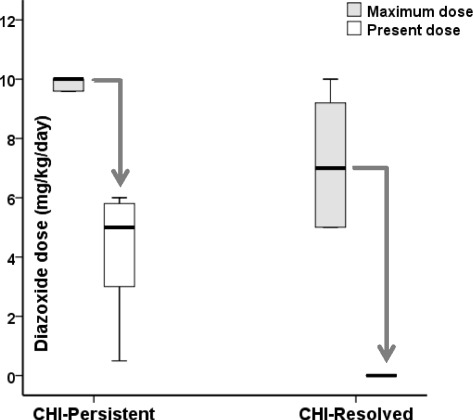
Maximum and present doses of diazoxide in children with CHI represented as *box* and *whisker plots* (median, 95% confidence intervals). In persistent CHI (CHI-Persistent), a higher maximal dose of diazoxide was required than in patients with resolved CHI (CHI-Resolved). Diazoxide dose was reduced both in CHI-Resolved and CHI-Persistent groups of patients

Fourteen patients (67%) received diazoxide treatment with good treatment response. Seven (33%) patients received SSRA treatment because they were either unresponsive or partially responsive to diazoxide (*n* = 6) or developed adverse reactions to diazoxide (*n* = 1). In follow up assessments, diazoxide dose was reduced in all patients [8.8(5.0, 15.0) to 0.0(0.0, 10.8) mg/kg/day (*p* < 0.001 for difference)] (Fig. 1). Eight patients on diazoxide achieved resolution after a period of 1.6(0.2, 13.0) years. Six patients on diazoxide did not achieve resolution and remained on treatment, although dose was reduced significantly [10.0 (8.5, 15.0) to 5.4 (0.5, 10.8) mg/kg/day, *p* = 0.003] after a period of 4.1 (1.1, 8.9) years. In 7 patients who received SSRA treatment [maximum octreotide dose 17.0(3.8, 19.2) mcg/kg/day], resolution was achieved in all. Resolution following SSRA treatment was noted in 2 patients (patients #11 and #15) who presented beyond the neonatal period. Patient #15 had diffuse CHI and was responsive to SSRA treatment, which was preferred in favour of sub-total pancreatectomy. In contrast, the diagnosis of focal CHI in patient #11 was delayed as initial genetic screening by Sanger sequencing of *ABCC8* exons did not find a mutation. The paternal *ABCC8* mutation (Table 2) was later identified as a splice site mutation, with focal CHI being confirmed by 18-fluoro-dopa PET-CT scanning. While focal lesionectomy was being planned, the patient’s medical management was reviewed; SSRA was stopped with satisfactory glucose measurements on a profile and a fast.

Resolution tended to be later in those receiving SSRA than in those receiving diazoxide [5.9(1.6, 9.0) v 1.6(0.2, 13.0) years of treatment, *p* = 0.08]. Overall, CHI resolved in 15 (71%) children in this cohort at age 3.1 (0.2, 13.0) years with age appropriate fasts in hospital (16–20 h) demonstrating absence of hypoglycaemia, suppressed insulin secretion and robust ketotic responses (Additional file 1: Figure S1 and Additional file 2: Figure S2) supported by satisfactory home glucose monitoring.

Factors associating with severity of illness were investigated for association with CHI resolution. GIR, a marker of severity of hypoglycaemia at presentation, was marginally less in resolved CHI than in persistent CHI patients [14.9(10.0, 18.5) v 16.5(13.0, 20.0) mg/kg/min, *p* = 0.6]. Maximum diazoxide dose was also significantly less in resolved CHI than in persistent CHI patients [6.0(5.0, 10.0) v 10.0(8.5, 15.0), *p* = 0.04]. Similar analysis was not performed in those on SSA, as resolution was achieved in all children.

### Neurodevelopmental outcomes

Mild delayed development was observed in 8 (38%) children in one or more domains (Table 1). The proportion of children having developmental delay was not significantly different between those with resolved CHI and persistent CHI [5(33%) v 3(50%), *p* = 0.5] and between those feeding orally and those requiring gastrostomy tube feeding [5(42%) v 3(33%), *p* = 0.7]. GIR was similar between those with and without developmental delay [15.7(13.0, 18.5) v 14.9(10.0, 20.0), *p* = 0.8]. Patients #9 and #17 with homozygous mutations and #12 with a compound heterozygous mutation had normal developmental outcomes. However patient #14 who had a homozygous mutation had mild motor and speech delay.

**Fig. 2:**
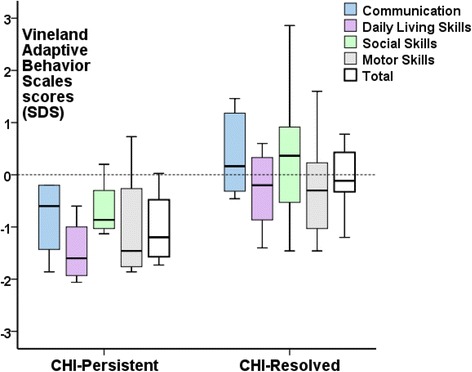
Vineland Adaptive Behavior Scales, 2nd edition (VABS-II) scores as standard deviation scores (SDS) for patients with persistent CHI (CHI-Persistent) and resolved CHI (CHI-Resolved), represented as *box* and *whisker plots* (median, 95% confidence intervals). Total SDS scores representing the Adaptive Behavior Composite (ABC) are shown in *white boxes* while individual domains are depicted in *colour*

VABS-II scores were available in 12 (57%) children older than 1.5 years of age (Fig. 2). VABS-II scores were within an acceptable population range at 0.3(−1.7, +1.6) SDS, with trend towards higher scores (better adaptive functioning) in resolved compared with persistent CHI [−0.1(−1.2, +1.6) v −1.2(−1.7, +0.1), *p* = 0.1] for most domains, but not reaching significance. Out of the VABS-II domains, daily living skills showed a significant difference with higher scores, i.e. a more favourable developmental outcome in resolved CHI compared to those with persistent CHI [−0.2 (−1.4, +0.6) v −1.6 (−2.0, −0.6), *p* = 0.02].

### Feeding outcomes

Twelve (57%) children were fed orally without requirement for nasogastric or gastrostomy tube feeding (Table 1). In those with oral food refusal and aversion, gastrostomy tube feeding continued in part or full for a variable period ranging between 1.3 and 5.1 years. Resolved CHI was similar in frequency between orally feeding and gastrostomy feeding children [8(67%) v 7(78%), *p* = 0.6]. Abnormal development was also similar in frequency between orally- and gastrostomy-fed children [5(42%) v 3(33%), *p* = 0.7].

## Discussion

Our study of young patients with K-ATP CHI suggests that resolution of CHI occurs in a significant proportion (71%) of those safely managed by conservative medical treatment. Resolution may not occur in all patients in prolonged follow up, but there is reduction in the intensity of treatment for hypoglycaemia, suggesting a trend of reducing severity of disease over time.

Our findings of reducing severity in both recessively or dominantly inherited *ABCC8/KCNJ11* mutations extend the recognised theme that dominant mutations may be mild [19] and that resolution can occur in a few children with recessively inherited disease [11, 20]. This notion is also commensurate with observations in large cohorts where patients with homozygous and compound heterozygous mutations may be medically managed without need for pancreatic surgery [7]. While it is recognised that the natural history of CHI may become clinically more manageable, our report provides objective and systematic evidence for this prevailing notion. Our findings also provide much needed prognostic information about the disease trajectory of K-ATP CHI and guidance for clinicians to re-evaluate severity at successive intervals and reduce medication as necessary.

We accept that patient numbers are relatively small and that only five patients with compound heterozygous and homozygous mutations represented severe diffuse medically treated CHI. However, patient numbers are not small for a rare disease drawn from a relatively large group of patients with genetic and non-genetic CHI over a 10 year period. Nonetheless, replication in other international cohorts would be helpful to prove the strength of association. Larger cohorts and international databases would be required to determine factors associated with reduction in severity as the number of patients in our cohort were too few (*n* = 7) to hypothesise mechanisms of disease resolution in CHI caused by recessively inherited mutations.

Only six children in this cohort remained on long-term medication. Two of these patients had missense mutations affecting *KCNJ11* residue p.R206. Three other patients tested in Exeter had mutations at this residue and had congenital hyperinsulinism that persisted for between 21 months and >3 years. The *ABCC*8 p.R526C mutation was reported in a patient who required treatment up to the age of 6 years [21]. However, a genotype:phenotype correlation is not absolute since the *ABCC8* p.I1512T mutation was found in another patient tested in Exeter whose hyperinsulinism remitted within a few days of birth.

In our study, we have provided genetic information on the type of K-ATP channel gene mutations in CHI patients. However, we have not investigated genotype predictions of natural history phenotype as *in-silico* predictions are unreliable in establishing pathogenicity and have not been tested in model predictions of disease trajectory. As experience in medical management of patients with K-ATP CHI accumulates worldwide, our study suggests the need to generate phenome databases to derive genotype-assisted prediction models of disease prognosis.

Although patients in our cohort had reducing severity, the neurodevelopmental phenotype was no different to previous cohorts [3, 5, 6]. This is likely to reflect adverse impact of hypoglycaemia in early life [3] and not likely to reflect the impact of continuing hypoglycaemia, as home glucose monitoring had been satisfactory in all patients. Further strength comes from the observation that the majority of the most severe patients, i.e. those with homozygous and compound heterozygous mutations had normal neurodevelopmental outcomes.

We did not observe deterioration in oral feeding with treatment reduction and disease resolution. The majority of children in this cohort were orally fed; those requiring gastrostomy tube feeding improved oral feeding over time. Therefore, treatment withdrawal or reduction was not associated with the collateral effect of increasing reliance on gastrostomy tube feeding.

Although we have reported a reduction in disease severity in the natural history and progression of genetic forms of CHI, we have been unable to find markers at presentation that could predict the resolution of disease. Therefore, it follows that CHI should be treated aggressively at the outset as recommended [1, 22], but with regular monitoring in follow-up to reduce treatment dosage, where feasible. The reduction in treatment intensity is not only a responsive management strategy, but also potentially reduces the significant harm to patients from excessive doses and prolonged exposure to medications with recognised toxic adverse effect profiles. We would recommend telephone and/or electronic communication every 2 weeks for the first 4 months to understand trends in home glucose profiles and drug response, followed by 4 monthly clinic reviews to assess the need for dose reduction. We would also suggest annual review of therapy for those remaining on treatment for longer than a year. Although we did not find patients experiencing relapse of hypoglycaemia in the relatively short duration of follow up, we would suggest on-going monitoring for the risk of hypoglycaemia, particularly during illness episodes for at least 2 years.

One criticism to adopt a step down treatment approach is the exposure to the potential risk of hypoglycaemia. However, the frequency of adverse neurodevelopment in our cohort was no different in those between resolution and persistence of CHI and no different than previous cohorts [5, 6]. The frequency of adverse neurodevelopment in the medically treated group has not been compared directly with the frequency in patients treated surgically in our cohort, although comparison of our data with other cohorts suggests a similar prevalence [4]. If early onset hypoglycaemia is the most important determinant of later life adverse neurodevelopment [3], it is unlikely that the small risk of hypoglycaemia from a proposed reduction in therapeutic intensity would be more detrimental. Nonetheless, it would be advisable to weigh up risks and benefits when offering treatment de-escalation choices to parents of children with CHI.

In our study of natural history outcomes, we did not evaluate glucose tolerance as part of the assessment of glycaemic outcomes, unlike other studies following pancreatectomy [23]. However, the utility of glucose tolerance testing at a young age in patients with resolving CHI not requiring surgery has not been established. Nonetheless, it would be important to evaluate formal glucose tolerance in older children and adolescents with resolved CHI to investigate the probability of evolving hyperglycaemia and diabetes.

## Conclusions

A reduction in severity of CHI was noted in all patients with K-ATP CHI, while a significant majority achieved hypoglycaemia resolution in follow up assessment, including those with compound heterozygous and homozygous mutations. Information about reducing severity could be discussed early in the management of CHI to guide prognosis and parental expectations. In children who are medically managed, disease severity should be periodically reviewed to assess the need to reduce medication dosage in anticipation of disease resolution.

## Additional files

Additional file 1: Figure S1. Mean blood glucose levels (95% confidence intervals) before and after prolonged fasting in patients with resolved CHI. (BMP 919 kb)
Additional file 2: Figure S2. Mean blood ketone (3 hydroxybutyrate) levels (95% confidence intervals) before and after prolonged fasting in patients with resolved CHI. (BMP 919 kb)

## Abbreviations

CGM: Continuous glucose monitoring
CHI: Congenital hyperinsulinism
EPA: Eicosapentaenoic acid
GIR: Glucose infusion rate
K-ATP CHI: Congenital hyperinsulinism due to mutations in K-ATP channel genes
PUFA: Polyunsaturated fatty acids
SDS: Standard Deviation Scores
SSRA: Somatostatin receptor agonists
VABS-II: Vineland Adaptive Behavior Scales, version II
