# Supplementary figures and images for: Conservatively treated Congenital Hyperinsulinism (CHI) due to K-ATP channel gene mutations: reducing severity over time
Source: Orphanet J Rare Dis. 2016 Dec 1;11:163. doi: 10.1186/s13023-016-0547-3 (PMC5133749; doi:10.1186/s13023-016-0547-3)

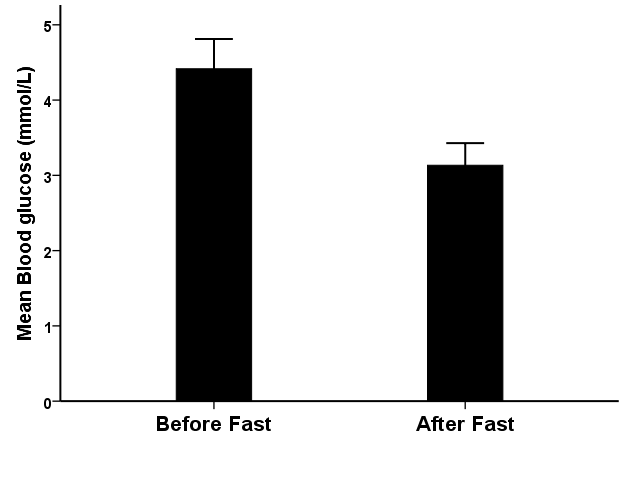

Supplement: Additional file 1: Figure S1. — Mean blood glucose levels (95% confidence intervals) before and after prolonged fasting in patients with resolved CHI. (BMP 919 kb) [file 13023_2016_547_MOESM1_ESM.bmp]

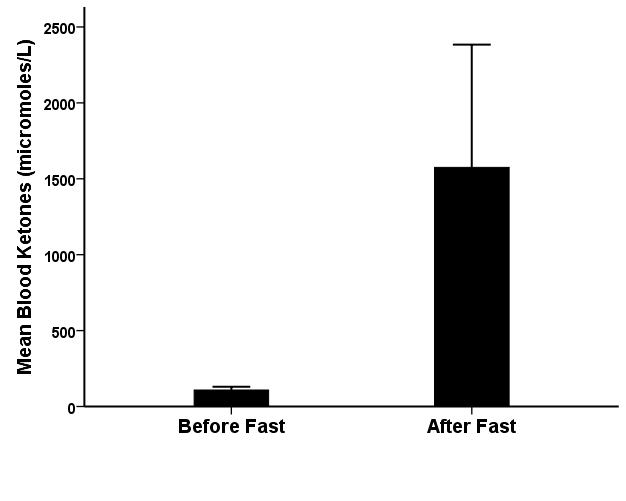

Supplement: Additional file 2: Figure S2. — Mean blood ketone (3 hydroxybutyrate) levels (95% confidence intervals) before and after prolonged fasting in patients with resolved CHI. (BMP 919 kb) [file 13023_2016_547_MOESM2_ESM.bmp]
